# Protective Effects of Dioscin against Lipopolysaccharide-Induced Acute Lung Injury through Inhibition of Oxidative Stress and Inflammation

**DOI:** 10.3389/fphar.2017.00120

**Published:** 2017-03-21

**Authors:** Hong Yao, Yiping Sun, Shasha Song, Yan Qi, Xufeng Tao, Lina Xu, Lianhong Yin, Xu Han, Youwei Xu, Hua Li, Huijun Sun, Jinyong Peng

**Affiliations:** ^1^College of Pharmacy, Dalian Medical University, DalianChina; ^2^Lab of Medical Function, College of Basic Medical Sciences, Dalian Medical University, DalianChina

**Keywords:** acute lung injury, dioscin, inflammation, lipopolysaccharide, oxidative stress, TLR4 signal pathway

## Abstract

The protective effects of dioscin, a natural steroidal saponin from some medicinal plants including *Dioscorea nipponica* Makino, against lipopolysaccharide (LPS)- induced acute liver and renal damages have been reported in our previous works. However, the actions of dioscin against LPS-induced acute lung injury (ALI) is still unknown. In the present study, we investigated the effects and mechanisms of dioscin against LPS-induced ALI *in vitro* and *in vivo*. The results showed that dioscin obviously inhibited cell proliferation and markedly decreased reactive oxidative species level in 16HBE cells treated by LPS. In addition, dioscin significantly protected LPS-induced histological changes, inhibited the infiltration of inflammatory cells, as well as decreased the levels of MDA, SOD, NO and iNOS in mice and rats (*p* < 0.05). Mechanistically, dioscin significantly decreased the protein levels of TLR4, MyD88, TRAF6, TKB1, TRAF3, phosphorylation levels of PI3K, Akt, IκBα, NF-κB, and the mRNA levels of IL-1β, IL-6, and TNF-α against oxidative stress and inflammation (*p* < 0.05). Dioscin significantly reduced the overexpression of TLR4, and obviously down-regulated the levels of MyD88, TRAF6, TKB1, TRAF3, p-PI3K, p-Akt, p-IκBα, and p-NF-κB. These findings provide new perspectives for the study of ALI. Dioscin has protective effects on LPS-induced ALI via adjusting TLR4/MyD88- mediated oxidative stress and inflammation, which should be a potent drug in the treatment of ALI.

## Introduction

Acute lung injury (ALI) often poses a great threat to human health (Bhatia and Moochhala, 2004; Rubenfeld et al., 2005). ALI is due to a large number of neutrophils into the lungs, and the release of a large number of pro-inflammatory mediators, resulting in damage to lung epithelial cells and endothelial cells (Sibille and Reynolds, 1990). Although significant progress has been made in the pathophysiology and treatment of ALI, the mortality rate remains unchanged (Steinberg et al., 2006). Therefore, it is urgent to improve the effective treatment strategy for the patients. The onset of ALI, an early symptom of organ failure, is associated with lipopolysaccharide (LPS) or the elevated blood levels of endotoxin (Jeyaseelan et al., 2004). Thus, LPS has been widely used to establish experimental model for drug development against ALI.

**FIGURE 1:**
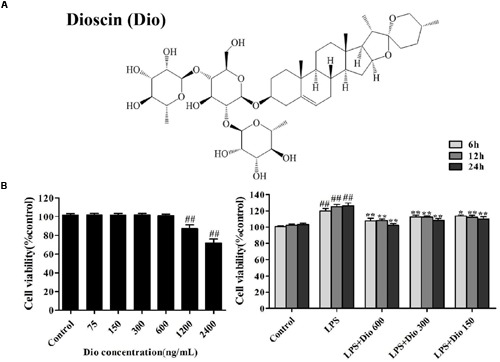
**Dioscin inhibits proliferation of the cells treated with lipopolysaccharide (LPS) *in vitro.* (A)** The chemical structure of dioscin. **(B)** Effect of dioscin (75, 150, 300, 600, 1200, and 2400 ng/ml) on the viability of 16HBE cells for 24 h, and the effect of dioscin (150, 300, and 600 ng/ml) on the proliferation of 16HBE cells treated with LPS (100 ng/ml) for 6, 12, and 24 h. Values are expressed as the mean ± SEM (*n* = 5). *p < 0.05 and **p < 0.01 compared with model group. ^##^*p* < 0.01 compared with control group.

In recent years, lots of work have been carried out to elucidate the mechanisms of LPS-induced ALI. However, the good therapeutic approach remains controversial and uncertain. Accordingly, there is an urgent need to develop effective drugs to treat this disease. Dioscin (Dio, **Figure 1A**), a natural saponin from some medicinal herbs (Dong et al., 2012), has anti-inflammatory, anti-tumor and anti-hyperlipidaemic effects (Kaskiw et al., 2009; Lu et al., 2011).

In our previous study, we have reported that dioscin has significant effects on l LPS-induced liver injury (Yao et al., 2016), LPS-induced kidney injury (Qi et al., 2016), non-alcoholic fatty liver disease (NAFLD) (Xu et al., 2014), hepatic ischemia- reperfusion damage (Tao et al., 2014), and hepatic fibrosis (Zhang et al., 2015). In our knowledge, dioscin has played significant roles on liver (Zhang et al., 2015; Yao et al., 2016), kidney (Qi et al., 2016), bone (Tao et al., 2016), and brain (Tao X.F. et al., 2015). Nevertheless, the effects and mechanisms of dioscin against LPS-induced ALI rmains un-known.

The purpose of our work was to test the actions of dioscin on ALI caused by LPS and then to elucidate the possible mechanisms.

## Materials and Methods

### Chemicals

Dioscin was prepared from *Dioscorea nipponica* Makino in our laboratory with the purity over 98% analyzed by high-performance liquid chromatography (Yin et al., 2010; Hu et al., 2013). Dioscin was dissolved with 0.1% dimethylsulfoxide (DMSO) for *in vitro* experiments, or with 0.5% carboxymethylcellulose sodium (CMC-Na) solution for *in vivo* tests. CMC-Na, 4’,6’-diamidino-2- phenylindole (DAPI), sodium dodecyl sulfate (SDS), and Tris were purchased from Sigma (St. Louis, MO, USA). A tissue protein extraction kit was obtained from Keygen Biotech. Co., Ltd. (Nanjing, China). A bicinchoninicacid (BCA) protein assay kit was purchased from the Beyotime Institute of Biotechnology (Jiangsu, China). MDA, SOD, NO, and iNOS assay kits were obtained from the Nanjing Jiancheng Institute of Biotechnology (Nanjing, China). RNAiso Plus, a PrimeScript^®^ RT Reagent Kit with gDNA Eraser (Perfect Real Time) and SYBR^®^ Premix Ex Taq^TM^ II (Tli RNase H Plus) were purchased from TaKaRa Biotechnology Co., Ltd. (Dalian, China).

### Cell Culture

The human bronchial epithelial (16HBE) cells were obtained from American type culture collection (ATCC) (Manassas, VA, USA) and maintaind in RPMI medium 1640 (Gibco, Carlsbad, CA, USA) with 10% fetal bovine serum (FBS) (Gibco, Carlsbad, CA, USA) in humidified atmosphere containing 5% CO_2_ and 95% O_2_ at 37°C.

### Dioscin Toxicity Assay

The 16HBE cells were seeded into 96-well plates at a density of 5 × 10^4^ cells/ml per well for 24 h before treatment, and then incubated for another 24 h in the presence of different concentrations of dioscin (75, 150, 300, 600, 1200, and 2400 ng/ml). The cell proliferation was measured using the MTT method.

### Cell Proliferation Assay

The 16HBE cells were plated into 96-well plates at a density of 5 × 10^4^ cells/ml for 24 h and then incubated for 6, 12, and 24 h in the presence of various concentrations of dioscin (150, 300, and 600 ng/ml) caused by LPS (100 ng/ml). The cells were measured according to the MTT method.

### Detection of Intracellular ROS Level

The 16HBE cells were plated in 6-well plates at a density of 5 × 10^4^ cells/ml and treated with dioscin at the concentrations of 150, 300 and 600 ng/ml for 24 h, then exposed to LPS for 24 h. The cells were harvested and re-suspended in 1 ml dichloro -dihydrofluorescein diacetate (DCFH-DA) (10 μM) for the detection of ROS level, which was imaged by fluorescence microscope (Olympus, Tokyo, Japan).

### LPS-Induced ALI *In vivo*

Male C57BL/6J mice weighing 18–22 g and male Wistar rats weighing 180–220 g were obtained from the Experimental Animal Centre of Dalian Medical University, Dalian, China (SCXK (Liao): 2013–0003). All experiments were approved by the Animal Care and Use Committee of Dalian Medical University, and the experimental procedures were performed in strict accordance with Legislation Regarding the Use and Care of Laboratory Animals of China. Before the experiments, the animals were allowed to suit the new environment for 7 days, and housed in a room under 12 h light/dark cycle, a controlled temperature at 23 ± 2°C and a relative humidity at 60 ± 10%. The mice and rats were randomly divided into five groups (*n* = 8 per group): control, model (LPS-treated) and dioscin-treated groups, respectively. The animals were oral administered with dioscin for 7 consecutive days at the doses of 80, 40, and 20 mg/kg for mice, and 60, 30, and 15 mg/kg for rats. Lung injury in mice and rats were induced by intraperitoneal (i.p.) LPS at the doses of 8 mg/kg and 5 mg/kg 2 h before the last administration. After 7 days, the animals were sacrificed after an overnight fast. Then, blood and lung tissue were collected and stored for further analysis.

### Histological Assay

The lung tissues were fixed in 10% formalin, embedded in paraffin, and sectioned into Five-micron-thick slices. The slices were then stained with haematoxylin-eosin (H&E). Images were captured using a light microscopy (Nikon Eclipse TE2000-U, Nikon, Japan) at 200× magnification, and the degree of lung injury was quantified using Image-Pro Plus 6.0 software.

### Antioxidant Assay *In vivo*

The levels of MDA, SOD, NO, and iNOS in lung tissues were detected using commercial kits according to the manufacturer’s instructions.

### Immunofluorescence Assay

For the immunofluorescence staining of TLR4 and MyD88, the tissue slices or formal in-fixed cells were preformed using primary antibodies (Santa Cruz, California, USA) in a humidified chamber at 4°C overnight. After washing twice in PBS, the tissue slices or the cells were incubated with a fluorescein-labeled secondary antibody for 1 h. Eventually, cell nuclei were stained with DAPI (5 μg/ml). The immunostained images were captured using a fluorescence microscope (Olympus, Tokyo, Japan).

### Quantitative Real-Time PCR Assay

Total RNA samples from lung tissues were extracted using RNAiso Plus reagent following the manufacturer’s protocol. Reverse transcription for cDNA synthesis and quantitative real-time PCR were performed as previously described. The forward (F) and reverse (R) primers for the tested genes are provided in **Table 1**. A no-template control was analyzed in parallel for each gene, and a GAPDH gene was selected as the housekeeping gene in our study. Eventually, the unknown template was calculated through the standard curve for quantitative analysis.

**Table 1:** The primer sequences used for real-time PCR assay in the present work.

Gene	GenBank accession	Forward primer (5’–3’)	Reverse primer (5’–3’)
Mouse GAPDH	NM_008084.2	TGTGTCCGTCGTGGATCTGA	TTGCTGTTGAAGTCGCAGGAG
Mouse TNF-α	NM_013693.2	TATGGCCCAGACCCTCACA	GGAGTAGACAAGGTACAACCCATC
Mouse IL-1β	NM_008361.3	TCCAGGATGAGGACATGAGCAC	GAACGTCACACACCAGCAGGTTA
Mouse IL-6	NM_031168.1	CCACTTCACAAGTCGGAGGCTTA	CCAGTTTGGTAGCATCCATCATTTC
Rat GAPDH	NM_017008.3	GGCACAGTCAAGGCTGAGAATG	ATGGTGGTGAAGACGCCAGTA
Mouse IL-1β	NM_031512.2	CCCTGAACTCAACTGTGAAATAGCA	CCCAAGTCAAGGGCTTGGAA
Rat IL-6	NM_012589.1	ATTGTATGAACAGCGATGATGCAC	CCAGGTAGAAACGGAACTCCAGA
Rat TNF-α	NM_012675.3	TCAGTTCCATGGCCCAGAC	GTTGTCTTTGAGATCCATGCCATT
Human GAPDH	NM_002046.3	GCACCGTCAAGGCTGAGAAC	TGGTGAAGACGCCAGTGGA
Mouse IL-1β	NM_000576.2	CTGAGCACCTTCTTTCCCTTCA	TGGACCAGACATCACCAAGCT
Human IL-6	NM_000600.3	TGGCTGAAAAAGATGGATGCT	TCTGCACAGCTCTGGCTTGT
Human TNF-α	NM_000594.3	TGTAGCCCATGTTGTAGCAAACC	GAGGACCTGGGAGTAGATGAGGTA

### Western Blotting Assay

The protein samples from the cells and lung tissues were isolated using the protein extraction kit (Beyotime Biotechnology, Haimen, China), and the protein content was determined using a BCA Protein Assay Kit. The protein samples were loaded on to the SDS-PAGE gel (8–15%), separated electrophoretically, and transferred on to a PVDF membrane (Millipore, USA). After blocking nonspecific binding sites for 1 h with 5 % dried skim milk in TTBS at 37°C, the membrane was individually incubated for overnight at 4°C with the primary antibodies listed in **Table 2**. Then the membrane was incubated at room temperature for 2 h with horseradish peroxidase-conjugated antibodies at a 1:2000 dilution. Protein expression was detected by the enhanced chemiluminescence (ECL) method. Protein bands were imaged using a Bio-Spectrum Gel Imaging System (UVP, Upland, CA, USA) and normalized with GAPDH as an internal control (IOD of objective protein versus IOD of GAPDH protein).

**Table 2:** The information of the antibodies used in the present work.

Antibody	Source	Dilutions	Company
GAPDH	Rabbit	1:2000	Proteintech Group, Chicago, IL, USA
TLR4	Rabbit	1:1000	Santa Cruz, CA, USA
MyD88	Mouse	1:1000	Santa Cruz, CA, USA
TRAF6	Rabbit	1:1000	Proteintech Group, Chicago, IL, USA
TKB1	Rabbit	1:1000	Proteintech Group, Chicago, IL, USA
TRAF3	Rabbit	1:1000	Proteintech Group, Chicago, IL, USA
p-PI3K	Rabbit	1:1000	Santa Cruz, CA, USA
PI3K	Rabbit	1:1000	Proteintech Group, Chicago, IL, USA
p-Akt	Rabbit	1:1000	Proteintech Group, Chicago, IL, USA
Akt	Rabbit	1:1000	Proteintech Group, Chicago, IL, USA
p-IκBα	Rabbit	1:1000	Proteintech Group, Chicago, IL, USA
IκBα	Rabbit	1:1000	Proteintech Group, Chicago, IL, USA
p-NF-κB	Rabbit	1:1000	Proteintech Group, Chicago, IL, USA
NF-κB	Rabbit	1:1000	Proteintech Group, Chicago, IL, USA

### TLR4 Gene Transfection in Cells

Overexpressed DNA transfection was used to upregulate TLR4 expression levels. The 16HBE cells were transfected with pPICZA-TLR4 plasmid DNA using Lipofectamine Plus Reagent (Invitrogen Life Technologies, CA, USA) according to the manufacturer’s instructions. Twenty-four hours after transfection, the cells were subjected to serum deprivation for 24 h before treated by LPS (100 ng/ml) in the presence or absence of dioscin (600 ng/ml) for an additional 24 h. Then, the level of ROS, and the protein levels of TLR4, MyD88, TRAF6, TKB1, TRAF3, p-PI3K, p-Akt, p-IκBα, and p-NF-κB were determined.

### Statistical Analysis

Data were presented as the mean ± standard error of the mean (mean ± SEM). One-way ANOVA or two tailed student’s *t*-test was used where appropriate. Statistical significance was set at *p* < 0.05 or *p* < 0.01.

## Results

### Dioscin Inhibits Proliferation of the Cells Treated with LPS *In vitro*

As shown in **Figure 1B**, dioscin at the concentrations of 75, 150, 300, and 600 ng/ml for 16HBE cells under 24 h treatment showed no statistically significant difference in cell viability. Compared with LPS group, dioscin at the concentrations of 150, 300, and 600 ng/ml under 6, 12, and 24 h treatment significantly changed cell viability. Under these conditions, dioscin effectively inhibited cell proliferation treated by LPS with time- and dose- dependent manners.

### Dioscin Rehabilitates LPS-Induced ALI

**FIGURE 2:**
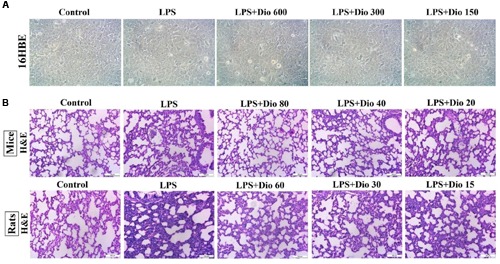
**Dioscin rehabilitates LPS-induced injury *in vitro* and *in vivo*. (A)** Effect of dioscin (150, 300, and 600 ng/ml for 24 h pretreatment) on the cellular morphology and structure of 16HBE by bright image (200 × magnification) investigation. **(B)** Effects of dioscin on LPS-induced lung injury in mice and rats based on H&E staining (200 × original magnification).

As shown in **Figure 2A**, dioscin at the concentrations of 150, 300, and 600 ng/ml under 24 h treatment was selected to protect LPS-induced ALI *in vitro*. As shown in **Figure 2B**, with the challenge of LPS, lung tissues were significantly damaged with the histopathologic changes including interstitial edema and hemorrhage, alveolar wall thickening, and notable infiltration of neutrophils and macrophages in the lung parenchyma and alveolar spaces. However, these symptoms were markedly reversed by dioscin.

### Dioscin Attenuates Oxidative Stress *In vitro* and *In vivo*

**FIGURE 3:**
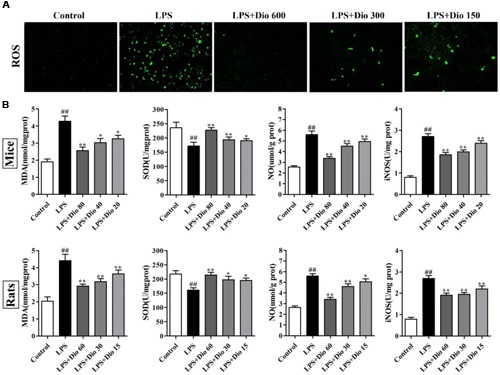
**Dioscin attenuates oxidative stress *in vitro* and *in vivo.* (A)** Effect of dioscin (150, 300, and 600 ng/ml for 24 h pretreatment) on the ROS level in 16HBE cells by immunofluorescence assay (200 × magnification). **(B)** Effect of dioscin on the levels of MDA, SOD, NO, and iNOS in mice and rats. Values are expressed as the mean ± SEM (*n* = 8). ^##^*p* < 0.01 compared with control group; *p < 0.05 and **p < 0.01 compared with model group.

As shown in **Figure 3A**, compared with model group, dioscin markedly decreased ROS level in 16HBE cells. As shown in **Figure 3B**, the levels of MDA, SOD, NO, and iNOS in mice and rats were significantly reversed by dioscin compared with model groups.

### Dioscin Adjusts TLR4/MyD88 Signal *In vitro*

**FIGURE 4:**
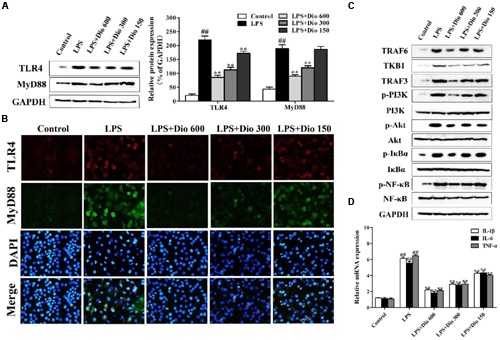
**Dioscin adjusts TLR4/MyD88 signal *in vitro*. (A)** Effect of dioscin on the protein levels of TLR4 and MyD88 in 16HBE cells. **(B)** Effect of dioscin on the expression levels of TLR4 and MyD88 in 16HBE cells based on immunofluorescence assay (400 × original magnification). **(C)** Effect of dioscin on the expression levels of TRAF6, TKB1, TRAF3, p-PI3K, p-Akt, p-IκBα, and p-NF-κB in 16HBE cells. **(D)** Effect of dioscin on the mRNA levels of TNF-α, IL-1β, and IL-6 in 16HBE cells. Values are expressed as the mean ± SEM (*n* = 3). ^##^*p* < 0.01 compared with control group; **p < 0.01 compared with model group.

As shown in **Figures 4A,B**, compared with model group, the protein levels of TLR4 and MyD88 in 16HBE cells were significantly down-regulated by dioscin in a dose-dependent manner based on western blotting and immunofluorescence assays. As shown in **Figure 4C**, the expression levels of TRAF6, TKB1, TRAF3, p-PI3K, p-Akt, p-IκBα, and p-NF-κB in model group were markedly increased compared with normal group, which were all significantly down-regulated by dioscin in 16HBE cells. The mRNA levels of IL-1β, IL-6 and TNF-α were also significantly decreased by dioscin *in vitro* (**Figure 4D**).

### Dioscin Adjusts TLR4/MyD88 Signal *In vivo*

**FIGURE 5:**
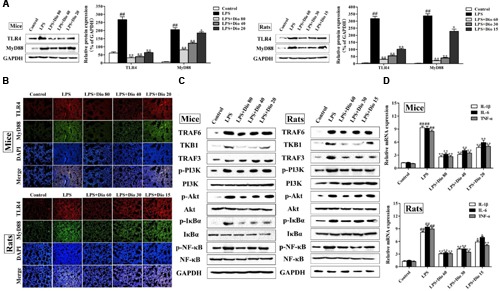
**Dioscin adjusts TLR4/MyD88 signal *in vivo.* (A)** Effect of dioscin on the protein levels of TLR4 and MyD88 in mice and rats. **(B)** Effect of dioscin on the expression levels of TLR4 and MyD88 based on immunofluorescence assay in mice and rats (200 × original magnification). **(C)** Effect of dioscin on the expression levels of TRAF6, TKB1, TRAF3, p-PI3K, p-Akt, p-IκBα, and p-NF-κB in mice and rats. **(D)** Effect of dioscin on the mRNA levels of TNF-α, IL-1β, and IL-6 in mice and rats. Values are expressed as the mean ± SEM (*n* = 3). ^##^*p* < 0.01 compared with control group; *p < 0.05 and **p < 0.01 compared with model group.

As shown in **Figures 5A,B**, compared with model groups, the protein levels of TLR4 and MyD88 in mice and rats were significantly down-regulated by dioscin in a dose-dependent manner based on western blotting and immunofluorescence assays. As shown in **Figure 5C**, the expression levels of TRAF6, TKB1, TRAF3, p-PI3K, p-Akt, p-IκBα, and p-NF-κB in model groups were markedly increased compared with normal groups, which were all significantly down-regulated by dioscin in mice and rats. The mRNA levels of IL-1β, IL-6, and TNF-α were also significantly decreased by dioscin (**Figure 5D**).

### TLR4 DNA Abrogates the Protective Effect of Dioscin

**FIGURE 6:**
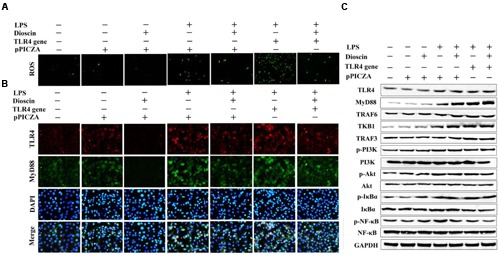
**TLR4 DNA abrogates the protective effect of dioscin. (A,B)** Effect of dioscin on the level of ROS, and the expression levels of TLR4 and MyD88 based on immunofluorescence assay (400 × original magnification). **(C)** Effect of dioscin on the protein levels of TLR4, MyD88, TRAF6, TKB1, TRAF3, p-PI3K, p-Akt, p-IκBα, and p-NF- κB in 16HBE cells after transfection.

As shown in **Figures 6A,B**, dioscin markedly suppressed ROS level, and the protein levels of TLR4 and MyD88 in cells were also decreased by the compound based on immunofluorescence assay. Compared with LPS group, the over-expressed level of TLR4 was down-regulated by dioscin after transfection. In addition, the protein levels of TLR4, MyD88, TRAF6, TKB1, TRAF3, p-PI3K, p-Akt, p-IκBα, and p-NF-κB were markedly decreased by dioscin after transfection (**Figure 6C**).

## Discussion

Acute lung injury is a direct and indirect damage caused by diffuse pulmonary interstitial and alveolar edema, and acute hypoxic respiration (Olleros et al., 2010; Singh et al., 2013). LPS, one ligand of TLR4 receptor, can induce the production of inflammatory cytokines and cause lung injury (Shi et al., 2013). There are evidences that TLR4 mediates microbial infection of immune and inflammatory responses and is involved in the pathogenesis of LPS-induced ALI (Coant et al., 2011; Jing et al., 2015). The TLR4 signaling pathway is involved in MyD88 dependent and non-dependent pathways, and the development of LPS-caused ALI may be one potent mediator to activate inflammation (Tao A. et al., 2015). The TLR4 signaling pathway includes a variety of downstream genes. Endogenous PI3K/Akt signaling pathway is one of the important downstream pathway, which can regulate the negative feedback of LPS stimulation (Ding et al., 2013). LPS activates TLR4/MyD88-dependent signaling pathways, leading to the phosphorylation of PI3K and Akt, which subsequently leads to nuclear translocation of NF-κB (Rana et al., 2015). NF-κB can regulate the levels of inflammatory cytokines including TNF-α, IL-6, and IL-1β (Gandoura et al., 2013).

Although inflammation is common in almost all patients with lung injury, the molecular link between inflammation and progression of lung injury and pneumonia remains unclear. Studies have shown that excessive release of ROS may lead to over activation of innate immune cells, overproduction of cytokines, and even the damage of the end organ in the process of LPS induced shock (Goodman et al., 2003; Krause and Bedard, 2008). TLR4 signal enhances ROS level, followed by the increased levels of MDA, iNOS, NO, and the reduced SOD level. High levels of SOD can protect ALI (Kong et al., 2010), and over-production of free radicals can increase MDA level (Powers et al., 2006). In the course of the development of ALI induced by LPS, the activation of pulmonary macrophages and inflammatory cell infiltration are the basic events of lung parenchyma damage (Abraham et al., 2006; Rittirsch et al., 2008). The excessive inflammatory response further leads to vascular injury and diffuse alveolar damage, which can aggravate lung injury and acute respiratory distress syndrome (Martin, 1997). For these reasons, these inflammatory mediators play a key role in ALI, and low levels of them may reduce LPS-induced ALI. As the main feature of inflammation in ALI, increased levels of inflammatory mediators can aggravate lung injury (Yeh et al., 2014). More importantly, LPS through TLR4 signal can produce proinflammatory cytokines to promote LPS-induced ALI (Imamura et al., 2009; Stoyanoff et al., 2014).

Dioscin has potent effects against LPS-induced liver and kidney injury in our previous studies (Qi et al., 2016; Yao et al., 2016). In our work, dioscin markedly protected LPS-induced 16HBE cell injury, and obviously alleviated the histopatholo- gical changes in mice and rats, suggesting that dioscin showed good action against ALI caused by LPS. We found that dioscin markedly suppressed the ROS level in 16HBE cells. In addition, PLS-caused high levels of MDA, NO, iNOS, and low level of SOD in mice and rats were all inhibited by dioscin, indicating that the anti-oxidant activity of the natural product may be one potential mechanism against LPS-induced ALI. What’s more, dioscin markedly decreased TNF-α, IL-1β, and IL-6 levels in mice and rats, suggesting that the anti-inflammatory effect of the natural product may be one potential mechanism against LPS-induced ALI. The mechanism results showed the action of dioscin on LPS-induced ALI may be via adjusting TLR4/MyD88 signal pathway.

## Conclusion

Our results demonstrated that dioscin conferred direct protective effects on LPS- induced ALI by inhibitiing oxidative stress and inflammation responses, which also provide new insights on the mechanisms of dioscin to treat ALI.

## Author Contributions

HY was responsible for the planning, execution of all experiments and preparation of the manuscript. YS, SS, YQ, and XT were responsible for LPS-induced acute lung injury model experiments. LX, LY, XH, and YX were responsible for the preparation, isolation and bioavailability study of dioscin. HL, HS, and JP provided critical inputs for the experiments. JP was responsible for the conceptualization, planning, execution and troubleshooting of the experiments, preparation of the manuscript and the financial support.

## Conflict of Interest Statement

The authors declare that the research was conducted in the absence of any commercial or financial relationships that could be construed as a potential conflict of interest.

## Abbreviations

Akt: protein kinase B
iNOS: inducible nitric oxide synthase
IκBα: inhibitor of nuclear factor κB alpha
IL-1: interleukin-1
IL-6: interleukin-6
MDA: malondialdehyde
MyD88: myeloid differentiation factor 88
NF-κB: nuclear factor κB
NO: nitric oxide
PI3K: phosphoinositide 3-kinase
SOD: superoxide dismutase
TKB1: TANK-binding kinase 1
TLR4: toll-like receptor 4
TNF-α: tumour necrosis factor-α
TRAF3: TNF receptor-associated factor 3
TRAF6: tumor necrosis factor receptor-associated factor 6.

## References

[B1] Abraham E. Nick J. A. Azam T. Kim S. H. Mira J. P. Svetkauskaite D. (2006). Peripheral blood neutrophil activation patterns are associated with pulmonary inflammatory responses to lipopolysaccharide in humans. *J. Immunol.* 176 7753–7760. 10.4049/jimmunol.176.12.7753 16751423

[B2] Bhatia M. Moochhala S. (2004). Role of inflammatory mediators in the pathophysio -logy of acute respiratory distress syndrome. *J. Pathol.* 202 145–156. 10.1002/path.1491 14743496

[B3] Coant N. Simon-Rudler M. Gustot T. Fasseu M. Gandoura S. Ragot K. (2011). Glycogen synthase kinase 3 involvement in the excessive proinflamm -atory response to LPS in patients with decompensated cirrhosis. *J. Hepatol.* 55 784–793. 10.1016/j.jhep.2010.12.039 21334395

[B4] Ding N. Zhang Y. Loughran P. A. Wang Q. Tsung A. Billiar T. R. (2013). TIFA upregulation after hypoxia-reoxygenation is TLR4- and MyD88-dependent and associated with HMGB1 upregulation and release. *Free Radic. Biol. Med.* 63 361–367. 10.1016/j.freeradbiomed.2013.05.029 23722163 PMC3752398

[B5] Dong H. Lu F. E. Zhao L. (2012). Chinese herbal medicine in the treatment of nonalcoholic fatty liver disease. *Chin. J. Integr. Med.* 18 152–160. 10.1007/s11655-012-0993-2 22311412

[B6] Gandoura S. Weiss E. Rautou P. E. Fasseu M. Gustot T. Lemoine F. (2013). Gene- and exon-expression profiling reveals an extensive LPS-induced response in immune cells in patients with cirrhosis. *J. Hepatol.* 58 936–948. 10.1016/j.jhep.2012.12.025 23321315

[B7] Goodman R. B. Pugin J. Lee J. S. Matthay M. A. (2003). Cytokine-mediated inflamma -tion in acute lung injury. *Cytokine Growth. Factor. Rev* 14 523–535. 10.1016/S1359-6101(03)00059-5 14563354

[B8] Hu M. M. Xu L. N. Yin L. H. Qi Y. Li H. Xu Y. W. (2013). Cytotoxicity of dioscin in human gastric carcinoma cells through death receptor and mitochondrial pathways. *J. Appl. Toxicol.* 33 712–722. 10.1002/jat.2715 22334414

[B9] Imamura M. Tsutsui H. Yasuda K. Uchiyama R. Yumikura-Futatsugi S. Mitani K. (2009). Contribution of TIR domain-containing adapter inducing IFN-beta-mediated IL-18 release to LPS-induced liver injury in mice. *J. Hepatol.* 51 333–341. 10.1016/j.jhep.2009.03.027 19501931

[B10] Jeyaseelan S. Chu H. W. Young S. K. Worthen G. S. (2004). Transcriptional profiling of lipopolysaccharide-induced acute lung injury. *Infect. Immun.* 72 7247–7256. 10.1128/IAI.72.12.7247-7256.2004 15557650 PMC529166

[B11] Jing W. Chunhua M. Shumin W. (2015). Effects of acteoside on lipopolysac -charide-induced inflammation in acute lung injury via regulation of NF-kappaB pathway in vivo and in vitro. *Toxicol. Appl. Pharmacol.* 285 128–135. 10.1016/j.taap.2015.04.004 25902336

[B12] Kaskiw M. J. Tassotto M. L. Mok M. Tokar S. L. Pycko R. Th’ng J. (2009). Structural analogues of diosgenyl saponins: synthesis and anticancer activity. *Bioorgan. Med. Chem.* 17 7670–7679. 10.1016/j.bmc.2009.09.046 19819703

[B13] Kong X. N. Thimmulappa R. Kombairaju P. Biswal S. (2010). NADPH oxidase -dependent reactive oxygen species mediate amplified TLR4 signaling and sepsis-induced mortality in Nrf2-deficient mice. *J. Immunol.* 185 569–577. 10.4049/jimmunol.0902315 20511556 PMC2913313

[B14] Krause K. H. Bedard K. (2008). NOX enzymes in immuno-inflammatory pathologies. *Semin. Immunopathol.* 30 193–194. 10.1007/s00281-008-0127-2 18560833

[B15] Lu B. N. Yin L. H. Xu L. N. Peng J. Y. (2011). Application of proteomic and bioinformatic techniques for studying the hepatoprotective effect of dioscin against CCl4-induced liver damage in mice. *Planta Med.* 77 407–415. 10.1055/s-0030-1250461 20979020

[B16] Martin T. R. (1997). Cytokines and the acute respiratory distress syndrome (ARDS): a question of balance. *Nat. Med.* 3 272–273. 10.1038/nm0397-272 9055847

[B17] Olleros M. L. Vesin D. Fotio A. L. Santiago-Raber M. L. Tauzin S. Szymkowski D. E. (2010). Soluble TNF, but not membrane TNF, is critical in LPS- induced hepatitis. *J. Hepatol.* 53 1059–1068. 10.1016/j.jhep.2010.05.029 20813418

[B18] Powers K. A. Szaszi K. Khadaroo R. G. Tawadros P. S. Marshall J. C. Kapus A. (2006). Oxidative stress generated by hemorrhagic shock recruits toll-like receptor 4 to the plasma membrane in macrophages. *J. Exp. Med.* 203 1951–1961. 10.1084/jem.20060943 16847070 PMC2118368

[B19] Qi M. Yin L. H. Xu L. N. Tao X. F. Qi Y. Han X. (2016). Dioscin alleviates lipopolysaccharide-induced inflammatory kidney injury via the microRNA let-7i/ TLR4/MyD88 signaling pathway. *Pharmacol. Res.* 111 509–522. 10.1016/j.phrs.2016.07.016 27431331

[B20] Rana M. Reddy S. S. Maurya P. Singh V. Chaturvedi S. Kaur K. (2015). Turmerone enriched standardized *Curcuma longa* extract alleviates LPS induced inflammation and cytokine production by regulating TLR4-IRAK1-ROS-MAPK- NFκB axis. *J. Funct. Foods* 16 152–163.

[B21] Rittirsch D. Flierl M. A. Day D. E. Nadeau B. A. McGuire S. R. Hoesel L. M. (2008). Acute lung injury induced by lipopolysaccharide is independent of complement activation. *J. Immunol.* 180 7664–7672. 18490769 10.4049/jimmunol.180.11.7664 PMC2753408

[B22] Rubenfeld G. D. Caldwell E. Peabody E. Weaver J. Martin D. P. Neff M. (2005). Incidence and outcomes of acute lung injury. *N. Engl. J. Med.* 353 1685–1693. 10.1056/NEJMoa050333 16236739

[B23] Shi H. Dong L. Jiang J. Zhao J. Zhao G. Dang X. (2013). Chlorogenic acid reduces liver inflammation and fibrosis through inhibition of toll-like receptor 4 signaling pathway. *Toxicology* 303 107–114. 10.1016/j.tox.2012.10.025 23146752

[B24] Sibille Y. Reynolds H. Y. (1990). Macrophages and polymorphonuclear neutrophils in lung defense and injury. *Am. Rev. Respir. Dis.* 141 471–501. 10.1164/ajrccm/141.2.471 2405761

[B25] Singh A. K. Tripathi Y. B. Pandey N. Singh D. P. Tripathi D. Srivastava O. N. (2013). Enhanced anti lipopolysaccharide (LPS) induced changes in macrophage functions by *Rubia cordifolia* (RC) embedded with Au Nan particles. *Free Radic. Biol. Med.* 65 217–223. 10.1016/j.freeradbiomed.2013.06.006 23774043

[B26] Steinberg K. P. Hudson L. D. Goodman R. B. Hough C. L. Lanken P. N. Hyzy R. (2006). Efficacy and safety of corticosteroids for persistent acute respiratory distress syndrome. *N. Engl. J. Med.* 354 1671–1684. 10.1056/NEJMoa051693 16625008

[B27] Stoyanoff T. R. Todaro J. S. Aguirre M. V. Zimmermann M. C. Brandan N. C. (2014). Amelioration of lipopolysaccharide-induced acute kidney injury by erythropoietin: involvement of mitochondria-regulated apoptosis. *Toxicology* 318 13–21. 10.1016/j.tox.2014.01.011 24561306

[B28] Tao A. Song J. Lan T. Xu X. Kvietys P. Kao R. (2015). Cardiomyocyte -fibroblast interaction contributes to diabetic cardiomyopathy in mice: role of HMGB1/TLR4/IL-33 axis. *Biochim. Biophys. Acta* 1852 2075–2085. 10.1016/j.bbadis.2015.07.015 26209013

[B29] Tao X. F. Qi Y. Xu L. N. Yin L. H. Han X. Xu Y. W. (2016). Dioscin reduces ovariectomy-induced bone loss by enhancingosteoblastogenesis and inhibiting osteoclastogenesis. *Pharmacol. Res.* 108 90–101. 10.1016/j.phrs.2016.05.003 27155058

[B30] Tao X. F. Sun X. C. Yin L. H. Han X. Xu L. N. Qi Y. (2015). Dioscin ameliorates cerebral ischemia/reperfusion injury through the downregulation of TLR4 signaling via HMGB-1 inhibition. *Free Radic. Biol. Med.* 84 103–115. 10.1016/j.freeradbiomed.2015.03.003 25772012

[B31] Tao X. F. Wan X. Xu Y. W. Xu L. N. Qi Y. Yin L. H. (2014). Dioscin attenuates hepatic ischemia-reperfusion injury in rats through inhibition of oxidative-nitrative stress, inflammation and apoptosis. *Transplantation* 98 604–611. 10.1097/TP.0000000000000262 25083618

[B32] Xu L. N. Wei Y. L. Dong D. S. Yin L. H. Qi Y. Han X. (2014). iTRAQ- based proteomics for studying the effects of dioscin against nonalcoholic fatty liver disease in rats. *RSC Adv.* 4:30704 10.1039/C4RA03948C

[B33] Yao H. Hu C. S. Yin L. H. Tao X. F. Xu L. N. Qi Y. (2016). Dioscin reduces lipopolysaccharide-induced inflammatory liver injury via regulating TLR4/ MyD88 signal pathway. *Int. Immunopharmacol.* 36 132–141. 10.1016/j.intimp.2016.04.023 27135544

[B34] Yeh C. H. Yang J. J. Yang M. L. Li Y. C. Kuan Y. H. (2014). Rutin decreases lipopolysaccharide-induced acute lung injury via inhibition of oxidative stress and the MAPK-NF-kappaB pathway. *Free Radic. Biol. Med.* 69 249–257. 10.1016/j.freeradbiomed.2014.01.028 24486341

[B35] Yin L. H. Xu L. N. Wang X. N. Lu B. N. Liu Y. T. Peng J. Y. (2010). An economical method for isolation of dioscin from *Dioscorea nipponica* Makino by HSCCC coupled with ELSD, and a computer-aided UNIFAC mathematical model. *Chromatographia* 71 15–23. 10.1365/s10337-009-1407-2

[B36] Zhang X. L. Han X. Yin L. H. Xu L. N. Qi Y. Xu Y. W. (2015). Potent effects of dioscin against liver fibrosis. *Sci. Rep.* 5:9713 10.1038/srep09713 PMC4389718 25853178

